# Canine “Sniffin’ Sticks” enable behavioural olfactometry to complement neurological examination in dogs

**DOI:** 10.1038/s41598-026-60466-1

**Published:** 2026-08-13

**Authors:** L. H. Hagemann, H. A. Volk, M. Prielipp, N. Power Guerra, A. Sabiniewicz, T. Hummel, S. Meller

**Affiliations:** 1 Department of Small Animal Medicine & Surgery, University of Veterinary Medicine Hannover, Hannover, Germany; 2 Center for Systems Neuroscience Hannover, Hannover, Germany; 3 Smell and Taste Clinic, Department of Otorhinolaryngology, Technical University of Dresden, Dresden, Germany

**Keywords:** Canine olfaction, Dog, Eugenol, Normosmia, Odour threshold, Health care, Medical research, Neurology, Neuroscience

## Abstract

Olfactory function assessment in dogs remains limited compared to human medicine. This study aimed to develop a canine version of “Sniffin’ Sticks” (canSSt) as a clinical behavioural olfactometry tool and to establish normative olfactory thresholds across sex, age, and cranial morphology in dogs. Eighty healthy client-owned dogs underwent clinical and neurological examination. A custom set of 15 felt pens containing serial tenfold eugenol dilutions (smell of cloves) was used to determine thresholds. Dogs were sequentially exposed to increasing concentrations. Behavioural responses were assessed by direct observation and independent video review. The test revealed high intra- and interobserver agreement (ICC 0.77–0.91). The mean threshold was dilution step 14.09 (SD ± 0.79), i.e., 8.71 ×  10^-14^ g/ml eugenol. Exploratory psychometric modelling produced a lower threshold (IC_50_) of 14.87 (95%CI 14.96–14.8), i.e., 1.43 × 10^- 14^ g/ml. Empirical data revealed no significant effects of sex, neuter status, or skull type. However, olfactory sensitivity increased from juveniles to adults and declined in seniors, paralleling human patterns. The canSSt proved to be a clinically feasible tool for assessing olfaction, with dilution step ≈ 14 representing a normosmic reference within the used 15-item setup. Additional dilutions can capture the full olfactory spectrum in dogs.

**Supplementary Information:**

The online version contains supplementary material available at 10.1038/s41598-026-60466-1.

## Introduction

Dogs heavily rely on their sense of smell as a vital tool for gathering information about their environment, identifying other conspecifics, animals, and family members, and navigating their surroundings^1,2^. The importance of olfaction in canine behaviour and cognition is well-documented, with smell playing a key role.

Humans have long exploited this pronounced olfactory ability. Dogs have been trained to detect and track down hunting game for centuries^3–5^ and can be deployed for searching missing persons, explosives, and drugs^6–8^. Recent studies have also demonstrated the suitability of dogs as diagnostic tools in medicine, such as real-time detection of Coronavirus Disease 2019 and other infectious and non-infectious conditions^1,9–11^.

It is well established that several diseases can affect the olfactory system and lead to hyposmia or anosmia in both humans^12–15^ and dogs^16–20^. Therefore, greater emphasis on the assessment of canine olfactory function in the veterinary setting can ultimately improve the quality of care provided to dogs. Despite the substantial relevance of the sense of smell in dogs, its importance is not reflected in current clinical practice. This is due to the difficulty of objectively assessing the sense of smell in animals – and thus also the olfactory nerve – in clinical and neurological practice. An assessment, if any, is often based solely on the owner’s observations of the dog’s searching, playing, and eating behaviour^21^.

In various studies, olfactory performance in dogs was determined using operant conditioning^22–24^, the measured time required by dogs to find hidden food samples^25,26^, or electroencephalographic olfactometry^27,28^. However, these test procedures often require complex equipment or prior training of the dogs, making them susceptible to training and motivation biases and providing only coarse forms of classification (e.g., success vs. failure in food or odour detection). In addition, some are partly influenced by visual factors. A recent study presented a food searching task for untrained dogs without the influence of visual stimuli. It underscored the pivotal role that olfaction plays in assessing cognition, particularly sensory recognition, in ageing pet dogs^29^.

In contrast, behavioural olfactometry provides a non-invasive means of assessing olfactory sensitivity and determining precise (ordinal or near-continuous) threshold concentrations for specific odourants. An odourant, quantifiable in intensity, is sequentially introduced at increasing concentrations. The dog’s behavioural response to these escalating scent levels is then meticulously analysed to determine its olfactory threshold^20,27^. In human medicine, a tool named “Sniffin’ Sticks” has gained popularity for its efficacy in determining olfactory thresholds and evaluating overall olfactory function^30,31^. Abrams et al. have shown that an adaptation of these commercially available “Sniffin’ Sticks”, originally designed for humans, can be effectively utilised for behavioural olfactometry in dogs. Their study insightfully demonstrated that dogs with Sudden Acquired Retinal Degeneration Syndrome (SARDS) not only loose their sight, but also have diminished olfactory capabilities compared to sighted dogs and visually impaired dogs that do not have SARDS^20^. The researchers in the study selected eugenol as the odourant for testing because of its known relative selectivity towards olfactory receptors^27^. In this context, it is important to note that other odourants might be irritating and stimulate trigeminal receptors as well^32–34^.

Assessing olfactory function quantitatively in dogs can be challenging, and although olfaction is one of the dog’s most important cognitive tools, it still lacks a standardised examination procedure in veterinary clinical settings. There is a need for reliable and feasible tools to evaluate canine olfaction in clinical practice. In humans, “Sniffin’ Sticks” are a well-established clinical tool^30,31^, but its use in dogs has been only demonstrated in the aforementioned SARDS study and has not been extensively validated^20^. The aim of this study was twofold: (1) to generate normative olfactory threshold data in privately-owned healthy dogs taking into account signalement (i.e., demographic) factors, such as sex, age, and cranial morphology for a conventional series of 15 dilution steps (10-fold) with eugenol, and (2) to develop the methodology of canine “Sniffin’ Sticks” (canSSt) for quantitatively assessing olfactory function in dogs, providing a valuable resource for veterinarians and researchers and contributing an important new tool to the repertoire of neurological examination in dogs.

## Materials and methods

### Dogs

One hundred and fifty privately-owned dogs were recruited via social media platform accounts (e.g., Facebook, Instagram) belonging to the University of Veterinary Medicine Hannover. Owners provided informed written consent prior to study commencement. Data on the owners and general information of the dogs (sex, age, breed) as well as their medical history were recorded and processed in accordance with relevant current data protection regulations. As the aim of the study was to generate normative data in healthy dogs, inclusion required the absence of any clinically relevant findings in the medical history, general clinical examination, neurological examination, and any further specialised examinations or assessments, including routine laboratory testing and the Canine Dementia Scale questionnaire. All dogs underwent general clinical and neurological examinations by a state-certified veterinarian. Dogs were excluded if any clinical deviation was identified in the medical history, general clinical examination, neurological examination, or any further specialised examination. Following the experiments, all dogs remained with and in the private ownership of their respective caregivers. The study was approved by the ethic and animal welfare committee of the University of Veterinary Medicine Hannover, Germany, and all methods and experiments were conducted in accordance with pertinent animal experiment guidelines (ARRIVE, PREPARE) as well as regulations such as the EU Directive 2010/63/EU and the German Animal Welfare Act (TierSchG).

### Olfactory threshold testing kit

An olfactory threshold testing kit was prepared in the Department of Small Animal Medicine and Surgery of the University of Veterinary Medicine Hannover three weeks before the testing phase. It included 15 felt-tip pens (brand Magiin, Shanghai, China), filled with eugenol (Sigma-Aldrich, ≥ 98%, St. Louis, MO, USA) as the test odourant and propylene glycol (Sigma-Aldrich, ≥ 99.5%, St. Louis, MO, USA) as solvent. Additionally, another pen filled only with propylene glycol was added as a blank sample. Pen number 1 (= dilution step 1) was filled with eugenol in its pure form of 6.52 M (1.07 g/ml) and number 2 to 15 were sequential tenfold dilutions each with propylene glycol, such that pen number 15 (= dilution step 15) contained the lowest concentration of 6.52 × 10^− 14^ M eugenol. The composition of the test kit was equivalent to the modified kit of “Sniffin’ Sticks” from a previous study^20^.

Identifying odour contamination can be challenging, as it is invisible and often imperceptible to humans, particularly within concentration ranges that remain detectable to dogs, given their exceptional olfactory sensitivity^19,22^. To prevent odour contamination as effectively as possible, the pens were prepared under standardised conditions in a state-of-the-art, quality-controlled laboratory using a fume hood. The volumetric pipettes were changed after each preparation of a dilution step, and precise laboratory practices were strictly followed, including the use of nitrile gloves and careful avoidance of sample spillages. After preparation, the pens were separately stored in glass test tubes with metal caps for each glass tube to ensure no smell cross-contamination.

### Test protocol

All assessments were conducted by the same examiner in the same standardised examination room under automatically controlled environmental conditions. Temperature, humidity, and continuous ventilation were maintained at constant levels (21.0–21.5 °C, humidity 35–50%) to minimise environmental effects on odourants and olfactory test outcomes^19,35,36^. The examination room had no natural daylight and was not used for any other procedures throughout the entire testing period. Testing was conducted in the morning, with six to eight dogs tested per session and a 20-minute interval between dogs. Between individual assessments, surfaces and tables were briefly cleaned using paper towels and water only, thereby avoiding potential odour contamination from cleaning agents. At the end of each testing day, the room was routinely cleaned and all surfaces were disinfected. Throughout the entire study period, the olfactory test kit was stored continuously in the same examination room under dark conditions. All capped pens were kept upright in their individual sealed glass tubes within a dedicated holder. To begin, each dog was allowed a 10-minute habituation period to acclimatise to the conditions of the testing room and the examiner to ensure undivided attention during testing. Dogs were handled by their owners and remained in close contact with them to minimise stress and ensure comfort. Small canine subjects were tested standing on an examination table, whereas large dogs were evaluated while standing on the ground. Subsequently, to eliminate collective visual stimuli that could skew the behavioural responses, all dogs were visually occluded and allowed additional 1–2 min of further habituation. Visual occlusion was achieved using individual, makeshift masks fashioned from compresses and plaster. To maintain consistency, the examiner wore nitrile gloves during the experimentation.

Initially, the blank sample was presented repeatedly until no behavioural patterns – possibly triggered by unfamiliar odours – were identified. Subsequently, the dilution steps (i.e., pen numbers) carrying different concentrations of eugenol were sequentially presented in ascending concentration order with each step (corresponding to descending dilution step number, starting at 15). Each dilution step, as well as the blank sample, was removed individually from the test kit while still contained within its glass tube. The metal cap of the glass tube was then removed, the pen was taken out, and the pen cap was opened carefully. The open pen was held approximately 2 cm in front of the dog’s nose for approximately 15 s, with discrete 15-second intervals between dilution steps. During each presentation, the pen was held carefully for an equal duration in front of the right as well as the left nostril, while avoiding abrupt air movements. After presentation, the pen was recapped, placed back into its glass tube, sealed, and returned in an upright position to the test kit holder, which was located approximately 1 m away from the dog during testing. This concentration-ascending series continued until the dog exhibited a distinct positive behavioural response, followed by several additional dilution steps. We defined a positive reaction as any sign of sniffing, withdrawal (responsive head or body movement, abrupt variations in breathing patterns), lip licking, ear twitching, swallowing, or barking, adapted from Abrams et al.^20^ and Myers & Pugh^27^. The dilution step, which initially drew a positive reaction, was documented and the test series ended. After a 5-minute break, the test was repeated for reliability in a second trial for each dog. Finally, the olfactory threshold was determined by taking the average of the documented dilution step numbers from both trials that first elicited a positive response. Supplementary Figure S1 provides a flowchart and a concise, practical guide and workflow intended to support clinicians in the application of canSSt in behavioural olfactometry.

### Evaluations

All tests were recorded using a video camera (4K, 60 frames per second, Pitiki, Yiwu, China), positioned in front of the dogs to allow further test evaluations. All recordings were evaluated by the first examiner and independently by a second observer, generating a total of 3 evaluations. Evaluation E_1_ (direct) and E_2_ (video) were assessed by the first observer with at least 12 weeks in between both evaluations. E_3_ was an assessment of the same videos by a second observer, who had no prior knowledge of the results from E_1_ and E_2_, allowing for both intra- and interobserver agreement analysis as well as for averaging all three evaluations (E_avg_).

### Data analysis

Microsoft Excel for Mac (version 16.103.3) was used for data capture, and the data were visualised or analysed using GraphPad Prism (GraphPad Software, Inc, version 10.4.1), Jeffreys’s Amazing Statistics Program (JASP, version 0.95.1), and BioRender.com. Descriptive data are presented as means with standard deviations (SD) in case of dilution steps, or geometric means with geometric SD factors in case of concentrations. Intra- and interobserver agreement was evaluated by assessing the intraclass correlation coefficient (ICC) with 95% confidence intervals (95%CIs). Paired t-tests were used to assess performance differences between trials. Predefined univariate subgroup analyses were performed to provide reference information for clinically relevant biological groups. Unpaired t-tests or one-way ANOVA were used to assess sex, age, and skull type differences in olfactory thresholds using E_avg_. To assess whether the observed subgroup patterns remained evident when the relevant biological variables were considered simultaneously, a multivariable linear regression analysis was performed using E_avg_ threshold values as the dependent variable and sex, age category, skull-type category, and neuter status as independent variables with 95%CIs. In addition, exploratory psychometric analyses with the same empirical data were conducted with response probability modelled as a function of concentration, in order to evaluate concentrations beyond the limited 15-item scale, with IC_50_ serving as the threshold concentration producing 50% of the predicted responses. For each dog, responses across trials and evaluations were aggregated as binomial proportions of detections per dilution step and transformed into cumulative detection probabilities with increasing concentration. The model assumes that each dog would also respond positively to all concentrations above its empirically determined threshold, irrespective of habituation effects, which were considered to play a subordinate role in the present context. The resulting mean cumulative response rates of dogs per dilution step were fitted with a four-parameter logistic (sigmoidal) function (bottom = 0, top = 1) using concentrations as predictors. 95%CIs were calculated for the mean cumulative response rates per dilution step as well as for the fitted curve and the IC_50_. For the full dataset, the IC_10_ as a meaningful model-based marker of the psychometric curve’s relevant onset was interpolated to determine a lower bound of the detectable responses within the extrapolated region (> dilution step 15). Extra sum-of-squares F-tests were conducted to compare IC_50_ values within defined sex, age, and skull type groups. All tests were two-sided and a p-value of less than 0.05 was considered significant.

## Results

### Dogs

Eighty privately-owned dogs fulfilled the predefined inclusion criteria and were included in the final cohort for olfactory threshold testing, whereas seventy dogs were excluded due to clinically relevant findings (Supplementary Table S1). The included population consisted of 35 male (22 intact; 13 neutered) and 45 female dogs (21 intact; 24 neutered). The 80 dogs (mean age ± SD = 4.3 ± 2.7 years, range 0.3–13 years) were divided into three age groups: Group 1 consisted of 24 juvenile dogs up to 2 years of age (mean ± SD = 1.45 ± 0.62 years, range 0.3–2 years), group 2 consisted of 46 adult dogs up to 8 years of age (mean ± SD = 4.54 ± 1.18 years, range 2.5–7 years) and group 3 consisted of 10 senior dogs aged 8 years or older (mean ± SD = 9.85 ± 1.56 years, range 8–13 years), according to Harvey^37^ and Bellows et al.^38^  The tested dogs comprised various breeds along with 30 mixed-breed dogs (Supplementary Figure S2). They were separated into a brachycephalic (*n* = 12) and meso-/dolichocephalic (*n* = 38) group based on the morphological descriptions in the Fédération Cynologique Internationale standards.

### Test evaluations and overall results

All 80 healthy dogs tolerated the visual occlusion well. Measured thresholds using the 15-step eugenol dilution series ranged from dilution step 15 (1.07 × 10^− 14^ g/ml) to dilution step 10 (1.07 × 10^− 9^ g/ml). The proportions and percentages of dogs responding at each dilution step across evaluations and trials are shown in Table 1. In none of the three evaluations were there significant differences between the two trials per animal (paired t-tests, *p* ≥ 0.439).

**Table 1:** Proportions and percentages of dogs responding to corresponding dilution steps across evaluations (E) and trials (T).

Dilution step	E_1_		E_2_		E_3_	
	T_1_	T_2_	T_1_	T_2_	T_1_	T_2_
15	28/80 35%	27/80 33.75%	40/80 50%	35/80 43.75%	46/80 57.5%	45/80 56.25%
14	25/80 31.25%	26/80 32.5%	23/80 28.75%	28/80 35%	19/80 23.75%	18/80 22.5%
13	19/80 23.75%	20/80 25%	13/80 16.25%	12/80 15%	11/80 13.75%	11/80 13.75%
12	6/80 7.5%	5/80 6.25%	2/80 2.5%	2/80 2.5%	2/80 2.5%	2/80 2.5%
11	1/80 1.25%	-	1/80 1.25%	-	1/80 1.25%	2/80 2.5%
10	1/80 1.25%	2/80 2.5%	1/80 1.25%	3/80 3.75%	1/80 1.25%	2/80 2.5%

Intra- and interobserver agreement were assessed using the ICC based on the average dilution step eliciting a positive response in E_1_ and E_2_ (same observer with direct or video assessment, respectively), and E_3_ (second observer analysing the same videos), yielding good to excellent results (ICC ≥ 0.77) (Table 2).

**Table 2:**
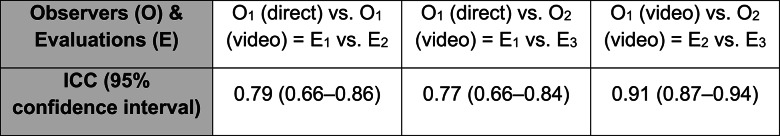
Independent intra- and interobserver agreement for olfactory threshold assessment by two observers with three evaluations.

Intraclass correlation coefficient (ICC): 0.9 to 1 (excellent agreement); 0.75 to 0.9 (good agreement); 0.5 to 0.75 (moderate agreement); below 0.5 (poor agreement); based on the average dilution step eliciting a positive response.

**Fig. 1:**
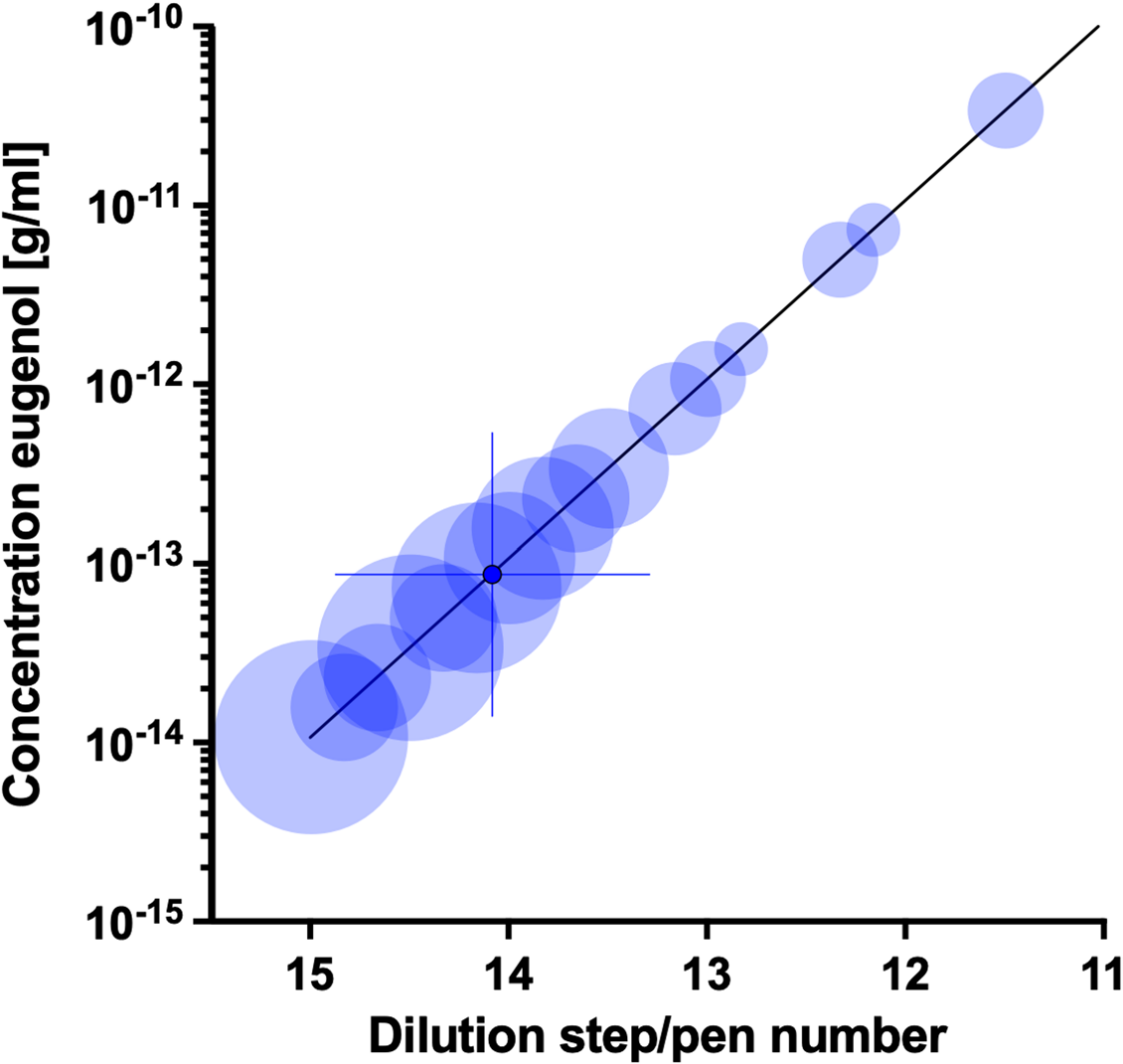
Empirically measured olfactory thresholds within the 15-dilution step (i.e., pen number) scale for the averaged evaluations (E_avg_) from 80 healthy dogs tested with eugenol. All measured threshold values fall along the reference line between dilution steps (x-axis) and corresponding concentrations (y-axis). The centres of the data bubbles represent the E_avg_ values along this line, and bubble size indicates the number of animals per value (smallest bubbles *n* = 1, largest bubble *n* = 13 at E_avg_ dilution step 15). The blue dot represents the arithmetic or geometric mean threshold for the dilution step and concentration scales, respectively, with error bars representing the standard deviation (SD) along the x-axis and the geometric SD along the y-axis.

The results of the mean olfactory thresholds for all three evaluations are shown in Table 3, with the overall E_avg_ yielding a mean threshold dilution step of 14.09 (SD ± 0.79) representing a geometric mean eugenol concentration of 8.71 × 10^− 14^ g/ml (geometric SD factor 6.18) within the 15-dilution step setup (Fig. 1). Due to strong observer agreement, all further analyses and data presentations were conducted with E_avg_.

**Table 3:**
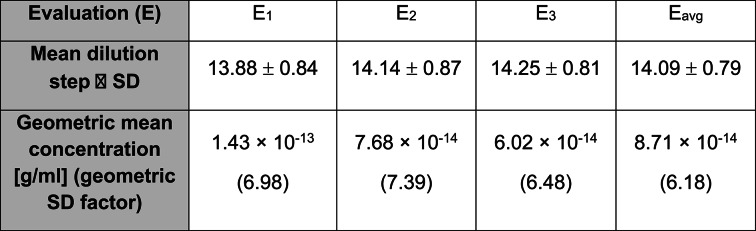
Evaluations and their average representing empirically measured mean olfactory thresholds on the used 15-dilution step eugenol scale across 80 healthy dogs.

Avg = average; SD = standard deviation.

### Effect of sex

**Fig. 2:**
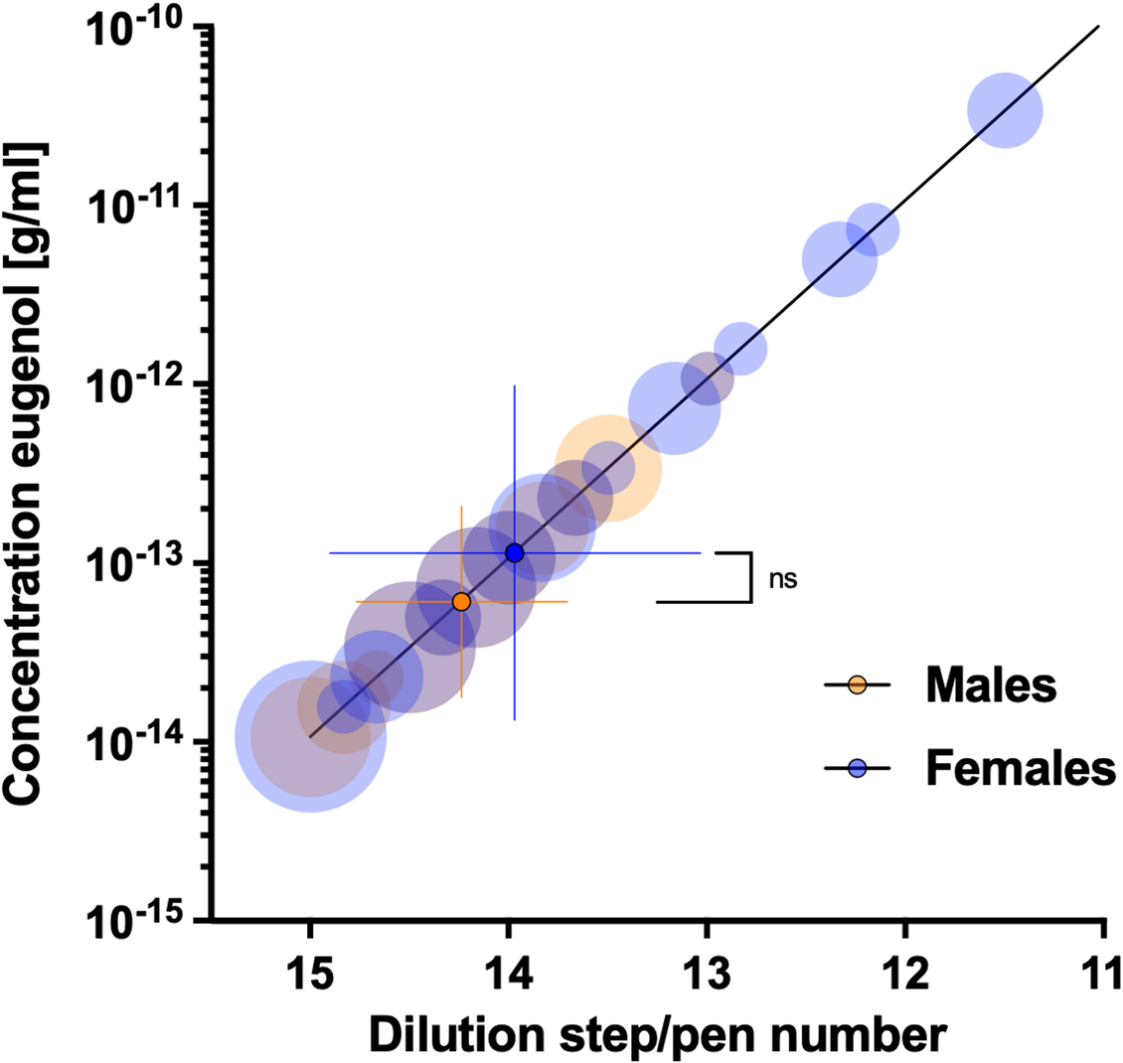
Empirically measured olfactory thresholds within the 15-dilution step (i.e., pen number) scale for the averaged evaluations (E_avg_) from 35 male and 45 female healthy dogs tested with eugenol. All measured threshold values fall along the reference line between dilution steps (x-axis) and corresponding concentrations (y-axis). The centres of the data bubbles represent the E_avg_ values along this line, and bubble size indicates the number of animals per value (smallest bubbles *n* = 1, largest bubble [females] *n* = 8 at E_avg_ dilution step 15). The dots represent the corresponding arithmetic or geometric mean threshold for the dilution step and concentration scales, respectively, with error bars representing the standard deviation (SD) along the x-axis and the geometric SD along the y-axis. Sex differences were not significant (ns; unpaired t-test).

Thirty-five male dogs exhibited a measured mean threshold dilution step of 14.24 (SD **±** 0.53) corresponding to 6.12 × 10^− 14^ g/ml eugenol (geometric SD factor 3.41). Fourty-five female dogs showed a mean threshold dilution step of 13.97 (SD **±** 0.93) corresponding to 1.15 × 10^− 13^ g/ml (geometric SD factor 8.57). No significant sex differences in thresholds were found (unpaired t-test; *p* = 0.127) (Fig. 2).

### Effect of age

**Fig. 3:**
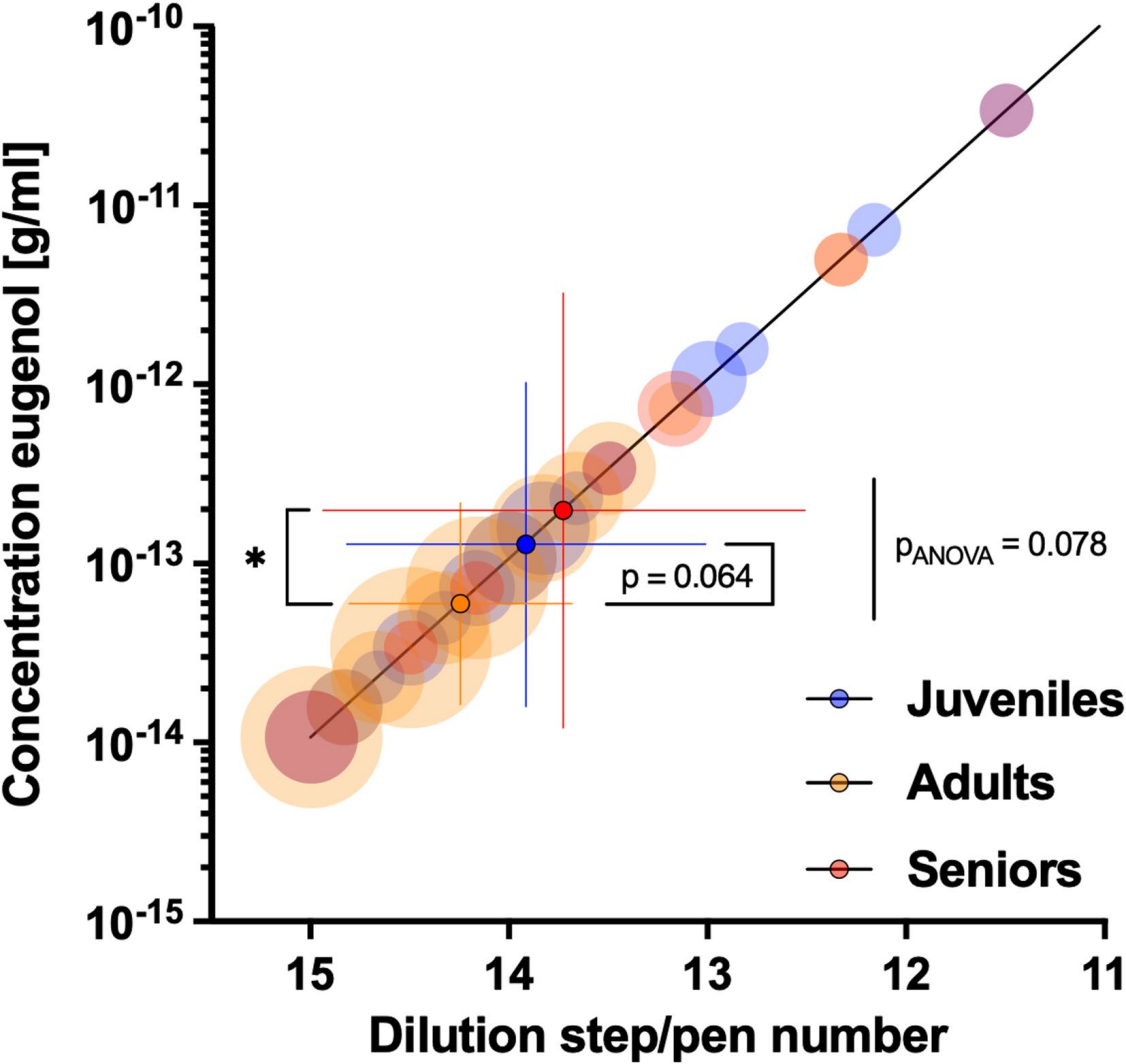
Empirically measured olfactory thresholds within the 15-dilution step (i.e., pen number) scale for the averaged evaluations (E_avg_) from 24 juvenile, 46 adult, and 10 senior healthy dogs tested with eugenol. All measured threshold values fall along the reference line between dilution steps (x-axis) and corresponding concentrations (y-axis). The centres of the data bubbles represent the E_avg_ values along this line, and bubble size indicates the number of animals per value (smallest bubbles *n* = 1, largest bubble [adults] *n* = 9 at E_avg_ dilution step of 14.5). The dots represent the corresponding arithmetic or geometric mean threshold for the dilution step and concentration scales, respectively, with error bars representing the standard deviation (SD) along the x-axis and the geometric SD along the y-axis. A one-way ANOVA showed a trending difference between groups (*p* = 0.078). Independent unpaired t-tests revealed a significant difference in thresholds between adult and senior dogs (* *p* ≤ 0.05) and a trend between adult and juvenile dogs (*p* = 0.064).

Juvenile dogs (*n* = 24) demonstrated a mean threshold dilution step of 13.92 (SD **±** 0.9), corresponding to 1.28 × 10^− 13^ g/ml eugenol (geometric SD factor 8.01). Adult dogs (*n* = 46) showed a mean threshold dilution step of 14.25 (SD **±** 0.56) corresponding to 5.97 × 10^− 14^ g/ml (geometric SD factor 3.65). Seniors (*n* = 10) showed a mean threshold dilution step of 13.73 (SD **±** 1.21), corresponding to 1.98 × 10^− 13^ g/ml (geometric SD factor 16.32). A one-way ANOVA only revealed a trend between age groups (*p* = 0.078). Independently conducted unpaired t-tests revealed a significant better olfactory performance in adults compared to senior dogs (*p* = 0.041) and a similar trend in adults compared to juvenile dogs (*p* = 0.064) (Fig. 3).

### Effect of skull type

**Fig. 4:**
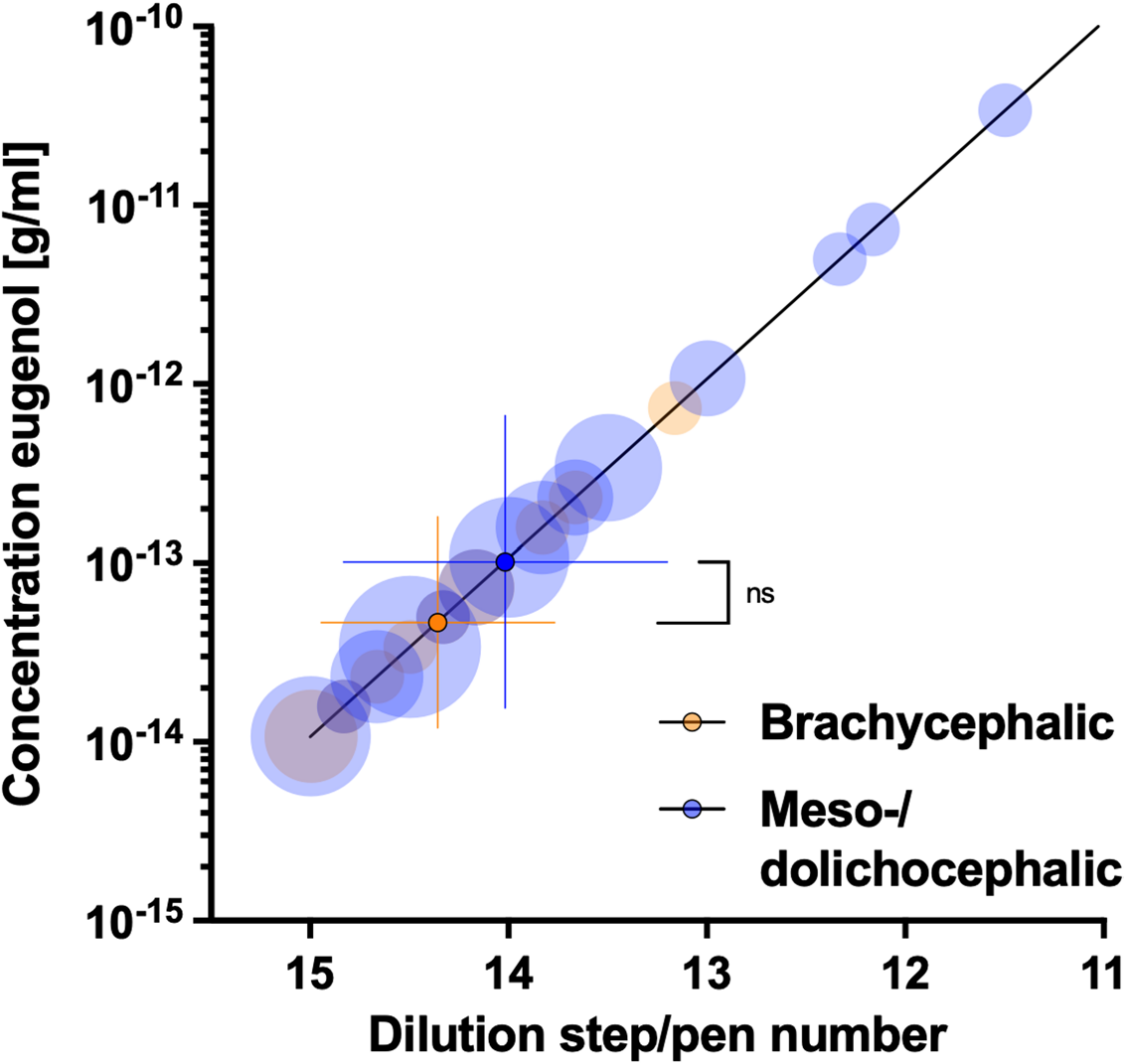
Empirically measured olfactory thresholds within the 15-dilution step (i.e., pen number) scale for the averaged evaluations (E_avg_) from 12 brachycephalic and 38 meso-/dolichocephalic healthy dogs tested with eugenol. All measured threshold values fall along the reference line between dilution steps (x-axis) and corresponding concentrations (y-axis). The centres of the data bubbles represent the E_avg_ values along this line, and bubble size indicates the number of animals per value (smallest bubbles *n* = 1, largest bubble [meso-/dolichocephalic] *n* = 7 at E_avg_ dilution step of 14.5). The dots represent the corresponding arithmetic or geometric mean threshold for the dilution step and concentration scales, respectively, with error bars representing the standard deviation (SD) along the x-axis and the geometric SD along the y-axis. No significant (ns) differences between groups were detected (unpaired t-test).

The group of brachycephalic dogs (*n* = 12) demonstrated an empirically assessed mean threshold dilution step of 14.36 (SD **±** 0.59), which corresponds to a eugenol concentration of 4.66 × 10^− 14^ g/ml (geometric SD factor 3.89). The group of meso-/dolichocephalic dogs (*n* = 38) showed a mean threshold dilution step of 14.02 (SD **±** 0.82) corresponding to a concentration of 1.02 × 10^− 13^ g/ml (geometric SD factor 6.54). Comparing the different skull types revealed no significant effect (unpaired t-test; *p* = 0.19). Thirty mixed-breed dogs were excluded from this analysis, as they could not be classified into the two groups (Fig. 4).

Finally, a complete-case multivariable linear regression analysis was performed in dogs with classified skull type (*n* = 50). The model was significant overall (F(5,44) = 2.516, *p* = 0.044). Senior dogs responded to significantly lower dilution step numbers (i.e., higher threshold values) compared with adult dogs after adjustment for sex, skull-type, and neuter status (β = −0.69, 95%CI: −1.32 to −0.05, *p* = 0.035), while juvenile dogs showed a similar but non-significant directional tendency (β = −0.4, 95%CI: −0.88 to 0.09, *p* = 0.110). Sex, skull-type category, and neuter status were not significantly associated with differing threshold values. However, neutered animals showed a tendency to respond at lower dilution step numbers than intact dogs, corresponding to higher olfactory thresholds in the former (β = −0.41, 95%CI: −0.85 to 0.03, *p* = 0.066).

### Exploratory psychometric models

**Fig. 5:**
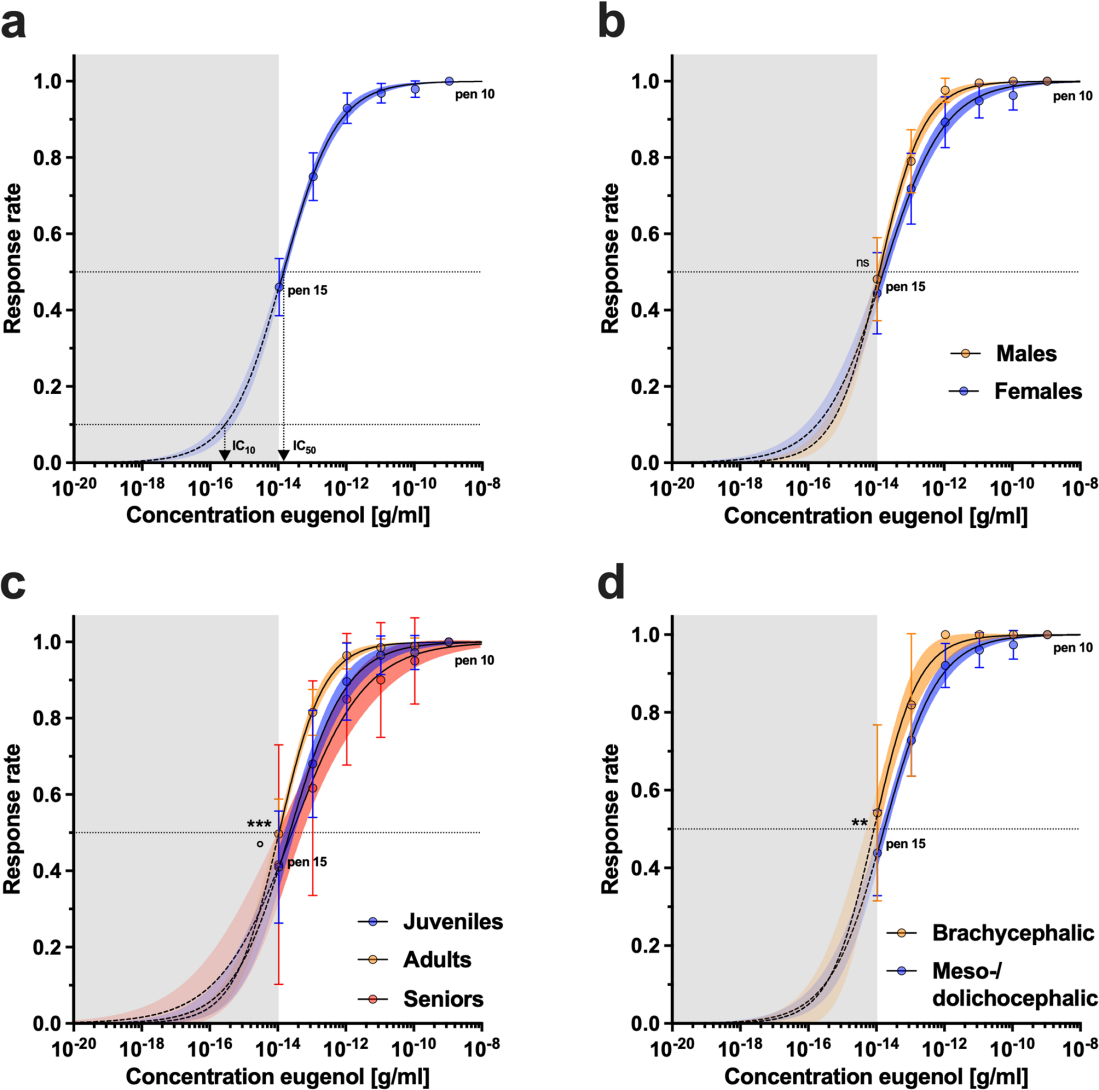
Exploratory modelled group-level olfactory thresholds (corresponding to IC_50_ = 50% of predicted responses) derived from the psychometric fit across measured thresholds from all evaluations and trials producing binomial response proportions per dog and presented dilution step from (**A**) all 80 healthy dogs, with subgroup analyses for (**B**) 35 male vs. 45 female dogs, (**C**) 24 juvenile vs. 46 adult vs. 10 senior dogs, and (**D**) 12 brachycephalic vs. 38 meso-/dolichocephalic dogs according to the Fédération Cynologique Internationale standards, tested with eugenol. The resulting mean cumulative response rates of dogs per dilution step with 95% confidence intervals (95%CIs; error bars) were fitted with four-parameter logistic (sigmoidal) functions (bottom = 0, top = 1) using concentrations as predictors (psychometric analysis). Shaded bands represent the 95%CIs of the fitted, independent curves. Please note that the models assume that each dog would also respond positively to all concentrations higher than its empirically determined threshold, irrespective of habituation effects, which play a subordinate role in the present research question. The modelled olfactory threshold IC_50_ for eugenol is represented as the concentration at which the fitted curve intersects the dashed horizontal line (long arrow in (**A**)). The dashed curves in the grey zone indicate extrapolations beyond the measured concentration range of the 15 dilution step system. The short arrow in (**A**) represents the interpolated IC_10_ value for all tested animals, serving as a reliable marker for a relevant onset of the overall curve. (**B**) No significant (ns) differences in model-derived IC_50_ values between sex subgroups were detected (F-test). (**C**) A global significant difference in model-derived IC_50_ values between curves was detected (*p* ≤ 0.001; F-test). Pairwise comparisons with F-tests revealed significant differences in IC_50_ values between adults and juveniles (*** *p* ≤ 0.001) as well as adults and seniors (° *p* ≤ 0.05). (**D**) A significant difference in model-derived IC_50_ values between skull type subgroups was detected (** *p* ≤ 0.01; F-test).

To complement and support the empirically measured thresholds beyond the 15-dilution step scale, exploratory psychometric functions were fitted to the response data from all evaluations and trials, with the modelled group-level olfactory threshold defined as IC_50_ (= concentration producing 50% of predicted responses) (Fig. 5). For the whole population, IC_50_ was at dilution step 14.87 with a 95%CI of 14.96–14.8, representing a modelled eugenol concentration of 1.43 × 10^− 14^ g/ml with a 95%CI of 1.19 × 10^− 14^–1.7 × 10^− 14^ g/ml (Fig. 5A). IC_10_ was interpolated to determine a lower bound of the detectable responses within the extrapolated region (> dilution step 15), yielding a virtual dilution step of 16.58, i.e. a concentration of 2.81 × 10^− 16^ g/ml for E_avg_ (Fig. 5A).

Psychometric analyses revealed a modelled group-level olfactory threshold (IC_50_) of 14.94 dilution steps (95%CI 15.03–14.85), i.e. 1.24 × 10^− 14^ g/ml (95%CI 1.01 × 10^− 14^–1.5 × 10^− 14^ g/ml) in males and 14.82 dilution steps (95%CI 14.95–14.7), i.e. 1.64 × 10^− 14^ g/ml (95%CI 1.2 × 10^− 14^–2.15 × 10^− 14^ g/ml) in females. Model-derived IC_50_ values did not differ significantly between the two datasets (F-test; *p* = 0.124) (Fig. 5B).

Psychometric analyses showed a modelled group-level olfactory threshold (IC_50_) of 14.68 dilution steps (95%CI 14.84–14.53), i.e. 2.25 × 10^− 14^ g/ml (95%CI 1.53 × 10^− 14^–3.13 × 10^− 14^ g/ml), in juveniles; 14.99 dilution steps (95%CI 15.07–14.92), i.e. 1.1 × 10^− 14^ g/ml (95%CI 9.12 × 10^− 15^–1.3 × 10^− 14^ g/ml), in adults; and 14.61 dilution steps (95%CI 15.03–14.3), i.e. 2.63 × 10^− 14^ g/ml (95%CI 1.01 × 10^− 14^–5.39 × 10^− 14^ g/ml), in seniors. A global F-test revealed that at least one IC_50_ value differed across the data sets (*p* = 0.0003). Pairwise F-tests for the IC_50_ among the three groups indicated that adults exhibited lower olfactory thresholds than juveniles (*p* = 0.0002) and seniors (*p* = 0.011) (Fig. 5C).

Psychometric analyses revealed a modelled group-level olfactory threshold (IC_50_) of 15.09 dilution steps (95%CI 15.31–14.93), i.e. 8.66 × 10^− 15^ g/ml (95%CI 5.19 × 10^− 15^–1.25 × 10^− 14^ g/ml) in brachycephalic dogs and 14.8 dilution steps (95%CI 14.92–14.7), i.e. 1.69 × 10^− 14^ g/ml (95%CI 1.29 × 10^− 14^–2.15 × 10^− 14^ g/ml) in meso-/dolichocephalic dogs. Model-derived IC_50_ values differed significantly between the two data sets (F-test; *p* = 0.005) with brachycephalic dogs showing lower modelled olfactory thresholds (Fig. 5D).

### Behavioural responses

**Fig. 6:**
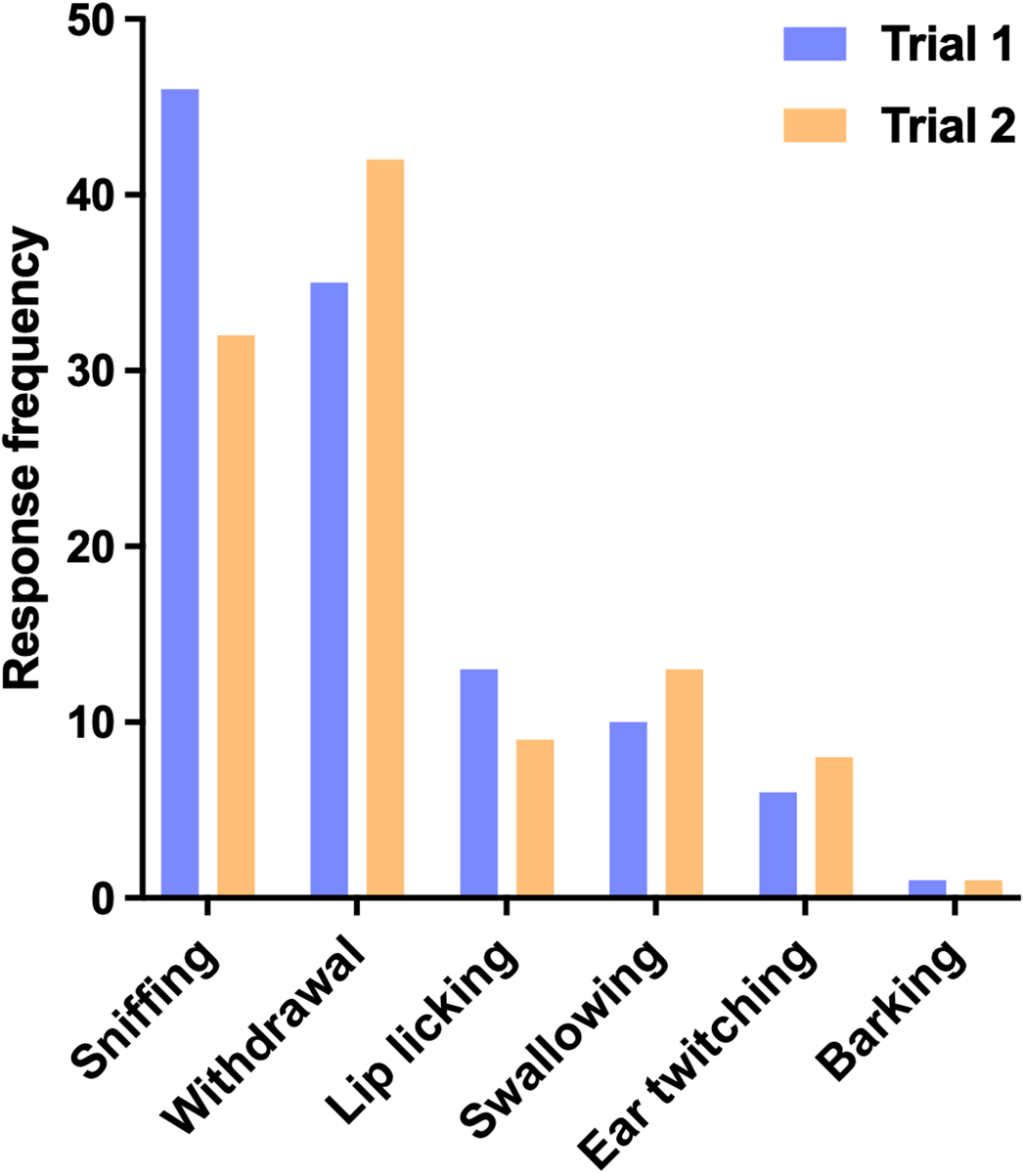
Descriptive and cumulative frequencies of observed behaviours towards the threshold-triggering dilution step across trial 1 (blue) and trial 2 (orange) based on the direct evaluation. When dogs (*n* = 80) displayed more than one behaviour, these were aggregated, resulting in a higher number of behavioural responses per trial than the number of dogs tested.

Type and frequency of behavioural responses that the dogs expressed towards the corresponding threshold dilution step in both trials were descriptively assessed based on the direct evaluation. The dogs showed either one or multiple signs simultaneously which were aggregated cumulatively as shown in Figure 6.

## Discussion

Despite the canine olfactory system’s role as an exceptional cognitive tool in dogs, neurological assessment of smell remains uncommon^39^. We adapted the human “Sniffin’ Sticks” method for canine behavioural olfactometry (canSSt) and assessed its feasibility for clinical settings^20,27,30,31^. Using 80 healthy, client-owned dogs, we generated normative reference values stratified by sex, age, and skull type. Taken together, these results support the adapted canSSt protocol as a practical, reproducible tool for clinical assessment of canine olfaction. Within the 15-dilution step configuration, a threshold dilution step of ≈ 14 (1.07 × 10^− 13^ g/ml eugenol) can serve as a reference for normosmia. As an additional exploratory, model-based level of analysis, the psychometric evaluation suggests extending the dilution series to at least step 18 (1.07 × 10^− 17^ g/ml) to capture the full performance range and avoid ceiling effects in testing. Sex‑ and age‑related aspects mirrored patterns reported in human cohorts.

Canine behavioural olfactometry is inherently limited by communication barriers and asymmetries in mutual understanding, requiring inference of odour detection from behavioural responses (Fig. 6) and being susceptible to non-olfactory cues such as vision or audition. In the current study, dogs tolerated visual occlusion with individually adjustable compresses, and we observed good to excellent intra- and interobserver agreement^40^ in line with prior studies^20,27^, thereby mitigating the risk of behaviour misinterpretation and supporting reliability. Threshold determination in untrained dogs hinges on the presence or absence of positive behavioural responses. Therefore, an oscillating staircase method as used in human olfactometry to fine-tune thresholds, with alternation between active affirmative and negative responses^30^, is not readily applicable. Instead, repeated trials with adequate breaks and fixed escalation protocols, starting from a common concentration until a positive response is observed, are necessary. No significant performance differences were observed between trials, consistent with the study from Abrams et al. showing high test-retest reliability^20^. Sniffing and withdrawal accounted for 72% of responses across trials, while other modalities were likewise clearly attributable to olfactory behaviour. Multiple blank sample presentations before each trial facilitate habituation and reduce noise in behavioural output. Nevertheless, increasing the number of trials will yield more robust conclusions as one of the main levers for aligning behavioural interpretation with actual odour detection.

The overall averaged analysis of eighty healthy dogs yielded a mean olfactory threshold dilution step of 14.09 (8.71 × 10^− 14^ g/ml eugenol; ≈ 84 ppq), consistent with Abrams et al., who reported a mean dilution step of 13.8 (17 ×  10^− 14^ g/ml eugenol) with the same setup^20^. The exploratory modelled psychometric IC_50_ threshold was slightly lower at dilution step 14.87 (1.43 × 10^− 14^ g/ml; ≈ 14 ppq) in the current study. Human thresholds for eugenol were measured at 6–30 ppb^41^ or 376 ppb^42^, respectively, i.e., five to seven orders of magnitude higher than in dogs. However, inter-study comparisons within or between species should be interpreted with caution due to methodological heterogeneity, differences in inclusion and exclusion criteria and sample size, as well as the substantial dimension of environmental factors, particularly in olfactory studies. Direct dog-human comparative studies using hexanal^43^, amyl acetate^44^, and α-ionone^45^ have reported differences in olfactory sensitivity of one (*in vitro*), 2.5, and three to four orders of magnitude, respectively.

Across species, amyl acetate and isoamyl acetate have been widely used in olfactory detection studies in dogs^22,28,44,46–49^, humans^44,50,51^, pigs^52^, and rodents^53,54^, enabling interspecies comparability. Although these compounds induce trigeminal or vomeronasal stimulation only at high intranasal concentrations^34,44,55,56^, we sought to isolate the trigeminal response as effectively as possible. We therefore chose eugenol, mostly unfamiliar to dogs and predominantly acting via the olfactory pathway. It shows minimal chemesthetic effects in humans with anosmia^32^ and is barely localised (i.e., lateralised between nostrils) at lower concentrations by normosmic subjects^57,58^, with localisation being a hallmark of trigeminal activation in humans^42,59^. On a functional level, Yousem et al. showed that eugenol primarily activates the orbitofrontal cortex, while trigeminally active compounds additionally elicit activation in the cingulate, temporal, cerebellar, and occipital regions^60^. (Iso-)amyl acetate shows similar broad activations, particularly in the insular cortex and cingulate regions, likely reflecting increased trigeminal input^56,61^. Although the purely olfactory effect of eugenol in humans is debated, especially at higher concentrations, its potential irritative mechanism appears complex. It depends on concentration and receptor stimulation dynamics, with even possible anaesthetic effects in the trigeminal system via sodium channel blockade^42,62,63^. Nevertheless, canine olfactory thresholds are so low, that trigeminal influence on threshold testing remains unlikely overall, irrespective of the compounds used.

For (iso-)amyl acetate, canine detection thresholds span from the ppm level down to to ≈ 1 ppt^22,28,44,46,49^. The wide range likely arises from methodological differences, including the presence/absence and design of training (e.g., duration, intervals, paradigms), the odour carriers and detection methods used (e.g., line-up, scent wheel), the number of sample containers, varying statistical approaches to threshold estimation, and the generally varying and small sample sizes (2–10 dogs). Moser et al. addressed this issue by using a habituation-dishabituation paradigm in 26 dogs, acclimating them to the carrier material first (habituation) before presenting successively increasing odourant concentrations (dishabituation), thereby assessing dogs’ investigation time for the novel odour and avoiding potential biases related to training and motivation^48^.

Our approach represents a modified habituation-dishabituation method that further reduces methodological and procedural biases by determining thresholds from dogs’ immediate behavioural responses rather than from arbitrary success rates or investigation times. It requires no prior training but relies on direct odourant exposure, enabling testing a large number of both healthy and diseased animals within a short period – an advantage particularly relevant for clinical and neurological applications. In the present study, dogs responded to eugenol concentrations below 84 ppq – lower than previously reported for (iso-)amyl acetate. Further comparative analyses are needed to determine whether dogs are indeed more sensitive to eugenol or whether these differences arise from methodological factors.

Notably, a 15-dilution step protocol with tenfold dilutions of eugenol, starting at 1.07 g/ml (= step 1), was used. To estimate detection limits beyond the 15-dilution range, exploratory psychometric models were built from empirical data, extending to lower concentrations. The overall mean threshold shifted from step 14.09 (empirical) to 14.87 (modelled, IC_50_). This is a minor change (≈ 0.78 dilution step units) of uncertain biological and clinical relevance, but clearly indicating that healthy dogs detect eugenol at concentrations below those covered by the 15-dilution step spectrum. Moreover, the model yielded an IC_10_ at dilution step 16.58 as a relevant initial parameter of the response curve, suggesting future studies or applications should extend dilutions of eugenol to step 17, 18, or beyond to empirically span broader ranges detectable by canine olfaction. However, modelling might yield some instability since the empirical values were mostly located in the upper part of the curves (Fig. 5). Consequently, these curves are intended primarily as a supportive measure. While dilution step 18 would correspond to 1.07 × 10^− 17^ g/ml (i.e., 0.01 ppq), Turunen et al. demonstrated a sensitivity in trained dogs down to ≈ 10^− 21^ for eucalyptol^24^.

The sample size of 80 dogs enabled analysis by basic signalment, i.e., demographic factors. Predefined univariate subgroup analyses were used to provide clinically interpretable reference information, while the multivariable regression analysis assessed whether these subgroup patterns remained evident when biological variables were considered simultaneously. Although some subgroups were small and partly imbalanced, both approaches yielded complementary findings overall and supported the observed patterns. No significant sex effects on eugenol thresholds were observed in the present study. Sex-specific differences in canine and human olfaction remain debated. Wei et al. reported higher densities and diversity of *c-fos*-positive and mitral cells in the olfactory bulb in female dogs linked to enhanced neuronal activity and long-time odour memory^64^. This suggests superior olfactory performance, paralleling findings that women generally outperform men, although effect sizes are typically small^30,65,66^. By contrast, Myers & Pugh also found no sex effect for eugenol in dogs^27^, and Nakaimuki et al. reported no sex-specific differences in olfactory functional connectivity in dogs using resting-state functional magnetic resonance imaging (fMRI)^67^. Sex differences in olfaction may also depend on the odourant type and its biological relevance^66^. While neutered dogs showed only a non-significant tendency towards higher olfactory thresholds in the present study, Salamon et al. likewise reported no discernible effect of neuter status on canine olfactory performance in a food searching task^68^. Overall, evidence regarding the influence of neuter status remains limited and inconsistent, and may depend on other or secondary factors such as dog characteristics and behaviour as well as the olfactory task used. Similarly, knowledge regarding the effects of reproductive and hormonal status on olfactory capabilities in dogs is scarce, although studies in other mammals, including humans, have demonstrated evident influences. For instance, a meta-analysis showed that olfactory sensitivity to specific odours appears to increase in women during fertile phases^69^, an effect that has also been observed in rodents^70,71^. These effects have been attributed, among other mechanisms, to changes in intrinsic functional and synaptic connectivity, as well as to the speed and complexity of processing in olfactory brain areas, and to direct hormonal modulation of olfactory neurons^69,72–74^. Effects of oestrous-cycle stage were not investigated in the present study.

Human studies consistently show an age-related decline in olfactory function^75–78^, likely due to degeneration of the olfactory epithelium and reduced number and function of olfactory sensory neurons (OSNs)^79,80^. Similar pathophysiological mechanisms are expected in dogs. Hirai et al. found a reduced number of OSNs and increased cerebrovascular amyloidosis in the olfactory bulb of dogs aged ≥ 14 years, suggesting decline in olfactory performance with age^81^. Nakaimuki et al. revealed a negative correlation between olfactory functional connectivity and age via resting-state fMRI^67^. Khan et al. demonstrated reduced olfactory performance in older dogs when locating hidden food samples. Some dogs were diagnosed with Canine Cognitive Dysfunction Syndrome based on cognitive testing and the Canine Dementia Scale (CADES) questionnaire^29^. To represent healthy ageing accurately, we excluded dogs with cognitive dysfunction. Our empirical and modelled data align with previously reported age-related findings. In addition, they reveal not only a decline in olfactory sensitivity from healthy adult to senior dogs but also suggest an increase from juvenile to adult dogs, based on a conventional age classification. This yields an inverted U-shaped curve for olfactory performance – rising in adulthood and declining in older age – similar to human data on odour threshold, discrimination, and identification^31,82^. Our findings may represent one of the first direct assessments of such an age-related threshold pattern in healthy dogs. This lifespan pattern is further supported by food-search and choice paradigms, where additional motivational and task-related factors may have an influence^29,68,83^. In line with these findings, Wei et al. reported higher neuronal activity in the olfactory bulb in adult dogs compared with younger individuals, which may indicate more efficient and complex processing of olfactory cues in mature animals^64^. Different cell types and circuits within the mammalian olfactory bulb continue to mature postnatally. The olfactory bulb, as well as other olfactory processing regions, remain subject to ongoing neurogenesis and synaptic plasticity^84,85^. Work in mice further highlights that the maturation and precision of projections from the olfactory epithelium to the olfactory glomeruli are highly dependent on odour exposure during the postnatal period^86^. In line with this, greater and broader odour experience, learning, and familiarity during development have been suggested to improve general olfactory performance in children and adolescents^87–90^. Together, these findings indicate that substantial postnatal development occurs in brain regions involved in olfactory processing and that exposure to odourants and odour-related experience may be important factors in the maturation of olfactory function. It cannot be excluded that behavioural immaturity or limited understanding of the testing procedure may mask olfactory performance in young individuals^91^. However, as described above, all animals tested in the present study tolerated the procedure well, and no obvious or pronounced difficulties in behavioural control during presentation of the dilution steps, such as excessive movement, were observed.

A central debate in canine research concerns the role of genetic, physiological, anatomical, and behavioural factors in breed-specific variation in olfactory performance^92,93^. Brachycephalic and meso-/dolichocephalic dogs differ considerably in anatomy and physiology, with brachycephalic breeds often showing respiratory tract issues suggesting possible olfactory impairments. Empirical evidence on this topic remains sparse and inconsistent to date^92^. A recent study found lower olfactory performance in dogs with rounder brains, but no clear link to skull type due to the composition of the study population. Brain shape was quantified as the neurocephalic index (brain width × 100/brain length) from dorsal- (i.e., axial-)plane MRI^67^. However, brachycephalic breeds potentially exhibit increased brain height in the dorsoventral direction^94^– a dimension not captured by the above formula and method for determining brain shape. Polymorphism in olfactory receptor genes and variation in pseudogenes may underlie breed-specific differences in olfactory performance as well^95^. However, recent work found no consistent genetic profiles in domestic dogs indicating higher sensitivity in particular breeds. Functional olfactory receptor gene repertoire size and relative cribriform plate size did not differ between skull types^96^. Superior olfaction in scent dog breeds appears linked more to behavioural traits and training rather than morphology or genetics^96^, though some studies reported better performance in selected sniffer dog breeds (e.g., German Shorthaired Pointer) compared to brachycephalic breeds (e.g., Boston Terrier)^26^. In contrast, others showed that brachycephalic Pugs discriminated odourants better than mesocephalic German Shepherds^97^. Notably, a recent study challenged these findings on a functional level indicating a degenerated olfactory receptor gene repertoire in Pugs^98^. In our empirical data, no significant difference in eugenol thresholds emerged between brachycephalic and meso-/dolichocephalic dogs, and the method used likely minimised training and motivation biases. Psychometric (IC_50_) modelling indeed indicated a lower threshold for brachycephalic breeds. However, as these are modelled values, this exploratory analysis should be interpreted with caution. Larger studies with broader concentration ranges are needed to provide more clarifying insights.

Limitations of the present study include the possibility that dogs may have experienced stress during spontaneous clinical testing, potentially biasing both the measurements and the behavioural interpretations. Owner presence and their continuous physical contact throughout the procedure, along with sufficient acclimatisation to the environment and examiner, were considered beneficial for maintaining the dogs’ calmness. Although eugenol, unlike in other regions, is not a typical odourant used in canine nose-work sports in Germany, general prior experience and training in scent work may have a potential effect on olfactory thresholds, which was not investigated in the present study. Both specific prior experience with individual odourants and general training status in scent work should be considered in future studies. Although the dilution steps were prepared under high-quality, state-of-the-art laboratory conditions using appropriate standardised procedures, the final concentrations were not independently analytically verified and should therefore be regarded as nominal concentrations. It should be noted that the interpretation of results from smaller subgroups, such as brachycephalic dogs (*n* = 12) and senior dogs (*n* = 10), should be made with caution. In addition, a purely anatomically based categorisation of skull shape, for example based on the cephalic index, was not conducted. This could have allowed the inclusion of mixed-breed dogs in the analyses and may have enabled a more detailed assessment. Such analyses and large-scale studies are needed to more conclusively determine the effect of skull shape on olfactory performance. While empirical and modelled data were comparable, future work should include additional dilutions or finer gradations to create a more reference-independent, higher-resolution dataset that captures thresholds beyond the given 15-dilution step spectrum used in the current study. Although the modelled data should be regarded as exploratory and interpreted with caution, they clearly illustrate this aspect. From a clinical perspective, however, the presented procedure is practical and appropriate given the effort-benefit ratio.

In conclusion, this study establishes the “Sniffin’ Sticks” test as a reliable, practical tool for assessing olfactory function in dogs in both veterinary practice and research, adapting a well-established method in human medicine. The adapted 15-item 10-fold dilution canine “Sniffin’ Sticks” (canSSt) protocol minimises training and motivation biases. It is feasible in terms of time, effort, sensitivity, and implementation, and – with high intra- and interobserver agreement – provides a robust tool for assessing olfactory function in dogs. Consistent olfactory behavioural responses from eighty healthy dogs yielded a normosmia threshold centred at dilution step ≈ 14 (i.e., ≈ 1.07 × 10^− 13^ g/ml eugenol), crucially within the 15-item reference framework. This framework should be extended to lower concentrations to capture an even broader spectrum of canine olfactory acuity, as suggested by modelled data. Threshold variations based on sex, age, or breed align with known patterns of olfactory performance in both dogs and humans, particularly regarding the age-related increase and subsequent decline in olfactory function. Thus, veterinarians can consider this method as an enrichment of the neurological examination, improving the standard of care in their practice and acknowledging the clinical importance of the dog’s most essential sensory and cognitive tool.

## Supplementary Information

Below is the link to the electronic supplementary material.

Supplementary Material 1

## Data Availability

The datasets generated during and/or analysed during the current study are available from the corresponding author on reasonable request.
