# Supplementary material for: Canine “Sniffin’ Sticks” enable behavioural olfactometry to complement neurological examination in dogs
Source: Sci Rep. 2026 Aug 13;16:25894. doi: 10.1038/s41598-026-60466-1 (PMC13487236; doi:10.1038/s41598-026-60466-1)
Supplement: Supplementary file 1 [file 41598_2026_60466_MOESM1_ESM.docx]

**Supplementary Material**

**Supplementary Table S1** Health conditions resulting in the exclusion of dogs

| **Primary disease or condition^1^** | **Proportions and percentages^2^** |
| --- | --- |
| Allergy | 5/70 (7.1%) |
| Aortic Stenosis | 1/70 (1.4%) |
| B-cell Lymphoma | 1/70 (1.4%) |
| Brachycephalic Obstructive Airway Syndrome | 4/70 (5.7%) |
| Canine Cognitive Dysfunction | 18/70 (25.7%) |
| Chronic Kidney Disease | 2/70 (2.9%) |
| Degenerative Myelopathy | 5/70 (7.1%) |
| Episcleritis | 1/70 (1.4%) |
| Hip Dysplasia | 4/70 (5.7%) |
| Hypoadrenocorticism | 1/70 (1.4%) |
| Hypoparathyroidism | 1/70 (1.4%) |
| Hypothyroidism | 5/70 (7.1%) |
| Leishmaniosis | 2/70 (2.9%) |
| Local Infection | 5/70 (7.1%) |
| Mild Trauma | 2/70 (2.9%) |
| Mitral Regurgitation | 1/70 (1.4%) |
| Nasal Tumor | 1/70 (1.4%) |
| Pancreatic Insufficiency | 1/70 (1.4%) |
| Patellar Luxation | 3/70 (4.3%) |
| Pulmonary Fibrosis | 1/70 (1.4%) |
| Seizures | 1/70 (1.4%) |
| Spondylosis | 4/70 (5.7%) |
| Ventricular Septal Defect | 1/70 (1.4%) |

^1^ Based on medical history, general clinical examination, neurological examination, and any further specialised examinations or assessments, including routine laboratory testing and the Canine Dementia Scale questionnaire. Please note that only the most relevant primary conditions are listed; some dogs may have had more than one secondary condition from the table or additional conditions not shown. ^2^ Percentages do not sum up to 100% due to rounding.

**Supplementary Figure S1** Action plan and workflow for behavioural olfactometry using canine „Sniffin’ Sticks“ (canSSt) for clinical applications. Blue boxes indicate the required actions, green boxes provide methodological details, and red boxes highlight relevant notes and information. Created in BioRender, including own digital manual tracing. M, S. (2026) https://BioRender.com/sxu6gts

**Supplementary Figure S2** Pie chart of 80 dogs stratified by breed and skull type. Created in https://BioRender.com
